# Modification and Evaluation of Avidity IgG Testing for Differentiating of Toxoplasma gondii Infection in Early Stage of Pregnancy

**Published:** 2013-08-24

**Authors:** Mohammad Reza Bonyadi, Parvin Bastani

**Affiliations:** 1 Infection Disease and Tropical Research Center, Immunology Research Center, Faculty of Medicine, Applied Research Center, Tabriz University of Medical Sciences, Tabriz, Iran; 2 Women’s Reproductive Health Research Center, Tabriz University of Medical Sciences, Tabriz, Iran

**Keywords:** Toxoplasmosis, Pregnancy, ELISA, IgG

## Abstract

**Objective:**

Toxoplasma gondii infection, an intracellular parasite, is often asymptomatic or is caused by different clinical diseases without being detected. Evaluation of IgG, IgA, and IgM in order to diagnose the pending Toxoplasmosis may confront some problems. Several researches has showned that Toxo IgG avidity can be useful in the recent active Toxoplasmosis. In current study, modification and importance of improved Toxoplasma Avidity IgG testing has been evlauated for differentiating Toxoplasma gondii infection in early stage of pregnancy.

**Materials and Methods:**

This experimental study included 300 pregnant women with risk of Toxoplasmosis in their initial months of pregnancy. We randomly divided 300 serum samples into A group (n=60) with high avidity and B group (n=40) with borderline avidity. The samples with Toxo IgG levels were classified to four subgroups. IgG avidity was evaluated by enzyme-linked immunosorbent assay (ELISA) method.

**Results:**

The mean absorbance of 100 samples in two groups was calculated, and then, two dilutaion curves with plotted absorbance against dilution were drawn for each serum sample. The results of this study showed that in groups with different concentrations of toxo IgG, appropriate dilution of serum is suitable for testing of Avidity. Our findings revealed the subgroups of 1, 2, 3, and 4 with serum dilutios of 1/3 , 1/6, 1/9, and 1/18 respectively, had real and good avidity.

**Conclusion:**

: One of the issues affectig the results of avidity is high concenteration of Toxo IgG in serum sample. As shown in this study, the best points of dilution for well avidity in both high and borderline avidities are marked with arrows in figures 1-8. This study confirmed that improved methods of measuring Toxo Avidity IgG are very important.

## Introduction

Toxoplasma gondii infection, an intracellular parasite, is often asymptomatic or causeed by different clinical diseases without being detected. However, parasite may be transmitted to the fetus after pregnant women are infected with the Toxoplasma gondii, so the fetus may be aborted or affected with severe neurological impairment and corioretinitis (1-3).

Evaluation of IgG, IgA, and IgM in order to diagnose the pending Toxoplasmosis may confront some problems. Serum IgG is valuable when the first test is negative, but the second one, after a few weeks, is positive. On the other hand, serum IgM remains positive for several months. Also, Rheumatoid factor (RF) causes false positive IgM. When the pregnant woman has high Toxo IgG and positive IgM, different tests must be perform for clarifying between a new infection or an old infection of Toxoplasma. In the cases with high levels of positive IgG and IgM before pregnancy, the embryo would be protected against Toxoplasma gondii infection, but in those cases with positive test result after pregnancy, the embryo is subjected to a serious risk. Initially, IgG avidity test has been applied through denaturalization technique for diagnosis of the congenital Rubellosis (4, 5).

In 1989, Hedman introduced a new method, named avidity test, which was based on the tendency of connecting immunoglobulin to Toxoplasma gondii polyvalent antigens using high density of urea to differentiate the high tendency of immunoglobulin. This test is recently used for detecting Toxoplasma IgG avidity (6). IgG avidity in initial steps of Toxoplasmosis infection is low, but the avidity of IgG from an old infection is very high. According to the recent researches, when the avidity is lower than 40%, it indicats initial infection or active steps, but when it is higher than 50%, it shows an old infection (7). In other studies, avidity titers lower than 30% has been reported as low avidity (8, 9). Increased titer of Toxoplasma IgG can also be used in recognition of recent Toxoplasmosis infection, but its monitoring requires a longer time that might be harmful to the embryo (10, 11).

Several researches have been shown that Toxo IgG avidity can be useful in the recent active Toxoplasmosis (7- 10). The timely diagnosis and treatment of Toxoplasmosis during pregnancy may protect the embryo from infection and consequent damages (12). Nowadays, regardless of high concentration of serum IgG antibodies against Toxoplasma gondii in avidity tests with commercial kits, a single sample of patient’s blood with high concentrations of urea is tested (4).

It seems that interference of high serum levels of IgG against Toxoplasma test results causes the negative outcomes. Our pilot research study showed both IgG Toxo concentration of 100 u /mL to 600 u /mL rise, the test problem is that it can have false negative results (Percent of avidity remains high). Therefore, if patient’s serum is diluted to different ratios before testing, appropriate dilution of the serum antibodies will be implemented and better results will be obtained.

In a comparative study of four commericaly available toxo avidity kits, one in three different dilutions of serum, while the other with single sample dilution were used, but it did not accurately determine that what dilutions of serum was the appropriate sample with no false negative result (13). In current study, modification and importance of improved Toxoplasma Avidity IgG testing has been evlauated for differentiating of Toxoplasma gondii infection in early stage of pregnancy.

## Materials and Methods

### Study design and population

This experimental study included 300 pregnant women in their initial months of pregnancy with risk of Toxoplasmosis between August, 2010 and August, 2012. We randomly divided 300 serum samples into A group (n=60) with high avidity and B group (n=40) with borderline avidity.

The women were referred to a reference Toxo avidity laboratory (Dr. Bonyadi’s lab, Tabriz, Iran). The result showed that both serum levels of Toxo IgG and IgM were positive. The avidity Toxo testing in patients was performed with single serum samples. The samples with Toxo IgG levels were classified to the following four subgroups: subgroup 1.100-199 Iu/ml, subgroup 2.200-299 Iu/mL, subgroup 3. 300- 399 Iu/mL, and subgroup 4.>400 Iu/mL.

### Enzyme-linked immunosorbent assay test

The levels of anti-toxoplasma IgG and IgM were measured at the beginning of pregnancy according to manufacturer’s instruction (Vircell Microbiology Co, Granada, Spain). The measurement of Toxo-IgG was quantitative, but that of Toxo-IgM was conducted through an index report by computing the cutoff point.

### Avidity test using enzyme-linked immunosorbent assay

Serial dilution from samples were prepared in the four series, including 1/3x, 1/6x, 1/9x, and 1/18x, which the "x" is the base dilution according to the kit instructions (In Vircel and Acone kit: x=20, while in Radim: x=100). For example, serial dilutions for Vircell and Acone kit are as follows: 1/60, 1/120, 1/180, and 1/360, while those for Radim Kit are as follows: 1/300, 1/600, 1/900, and 1/1800. A total of 400 tests were carried out according to Headmen’s method. Headmen’s method includes urea with concentration of 6 Mol placed in an incubation for 10 minutes, while one sample with urea and other sample without urea following the formula. (4). Patient’s diluted sera were added to micro plates coated with Toxoplasma antigen. In the second step, concentrated (8M) urea solution was added to the antigen-antibody mixture. After washing excess antibody, labeled anti-IgG antibody was added to the test micro plates. After 30 minute of incubation and re-washing, substrate solution was added, and in the final step, the reaction was stopped by adding sulfuric acid. The optical density (OD) was measured at 450 nm against the differential wavelength of 600 nm. The avidity was calculated by the following formula: Avidity Index (%)=(OD sample treatment with urea-OD blank)/(OD sample treatment without urea)×100.

The average light absorption of 100 samples with high and borderline avidity in different groups was plotted.

### Statistical analysis and ethical considerations 

Statistical analysis was performed by SPSS software package version 16.0 for windows (SPSS Inc., Chicago, USA). Quantitative data were presented as mean ± standard deviation (SD), while qualitative data were demonstrated as frequency and percent (%). For statistical analysis, collected data were studied using descriptive statistical methods. The study protocol was approved by the Ethics Committee of Tabriz University of Medical Sciences (TUMS), which was in compliance with Helsinki Declaration. All patients signed the consent in order to participate in this study

## Results

The average light absorption of each subgroup in four different serum dilutions and in two high and borderline avidity groups were calculated. All results were shown in figures 1-8.

The results of this study showed that in the groups with different Toxo IgG concentration, specific dilution of serum is suitable for avidity testing. For example in subgroups of 1-4, serum dilutions of 1/3, 1/6, 1/9, and 1/18, respectively, illustrate a real and perfect avidity.

**Fig 1:**
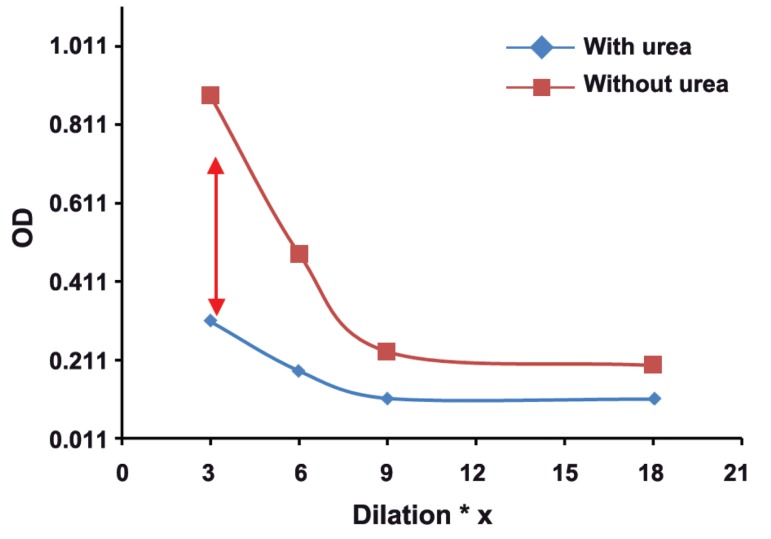
High avidityToxo IgG 100-199 Iu/mL.

**Fig 2:**
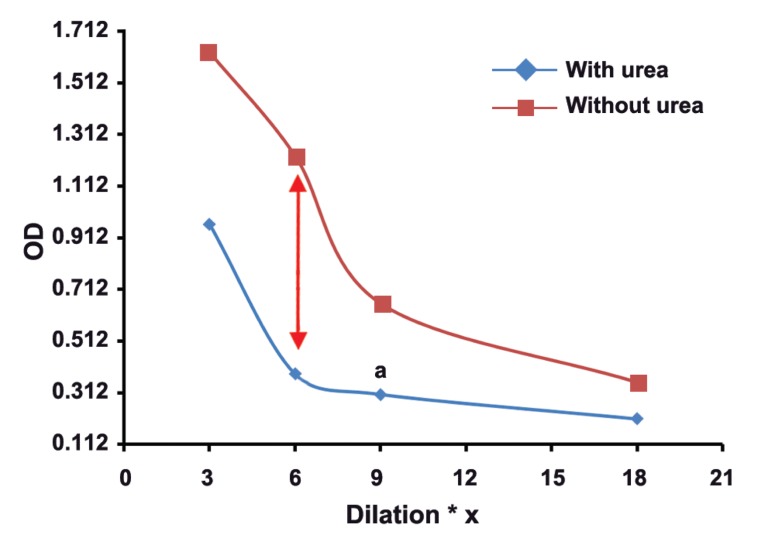
High avidityToxo IgG 200-299 Iu/mL

**Fig 3:**
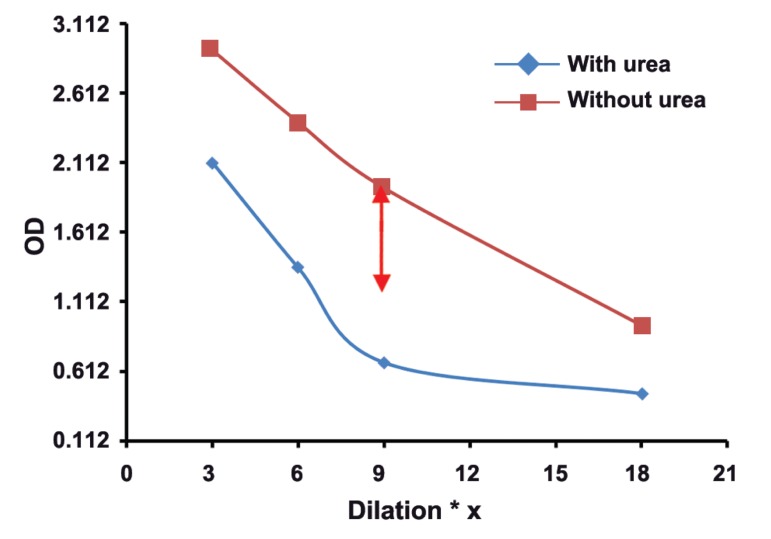
High avidityToxo IgG 300-399 Iu/mL

**Fig 4:**
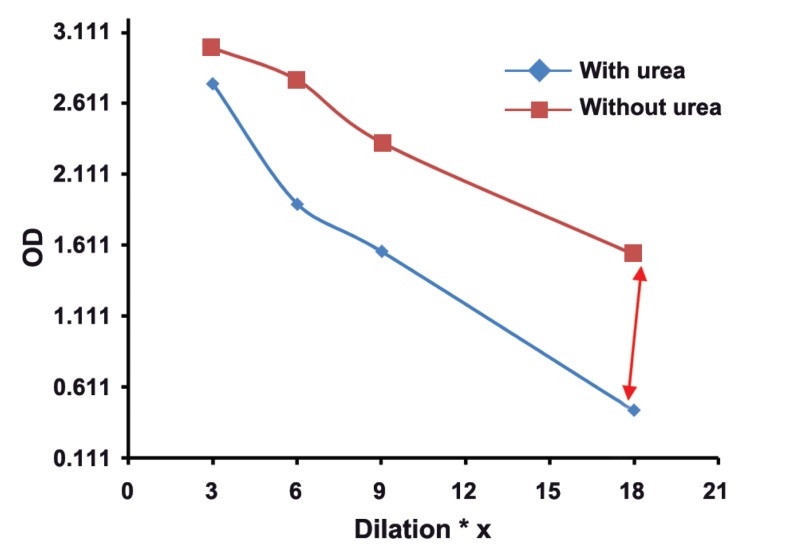
High avidityToxo IgG >400 Iu/mL.

**Fig 5:**
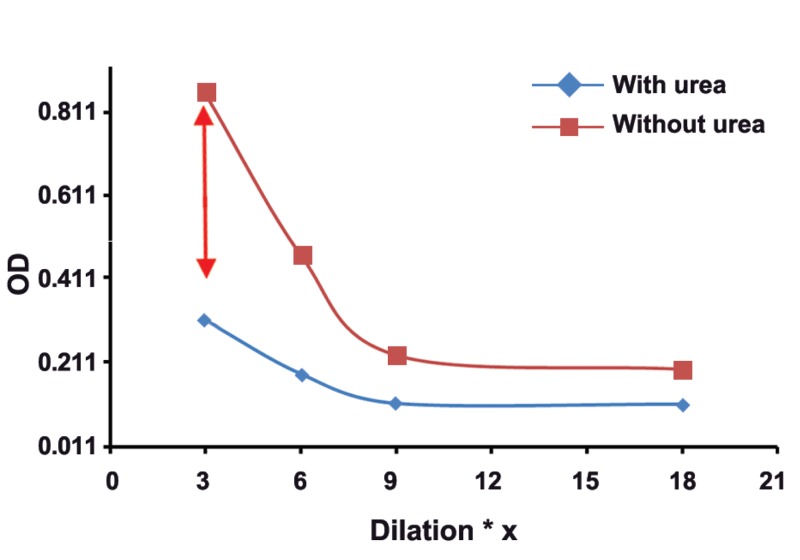
Borderline avidity Toxo IgG 100-199 Iu/mL.

**Fig 6:**
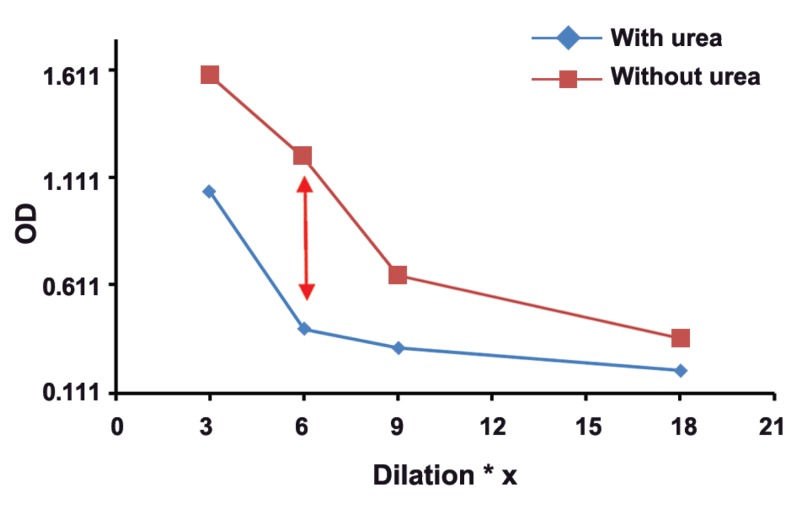
Borderline avidityToxo IgG 200-299 Iu/mL.

**Fig 7:**
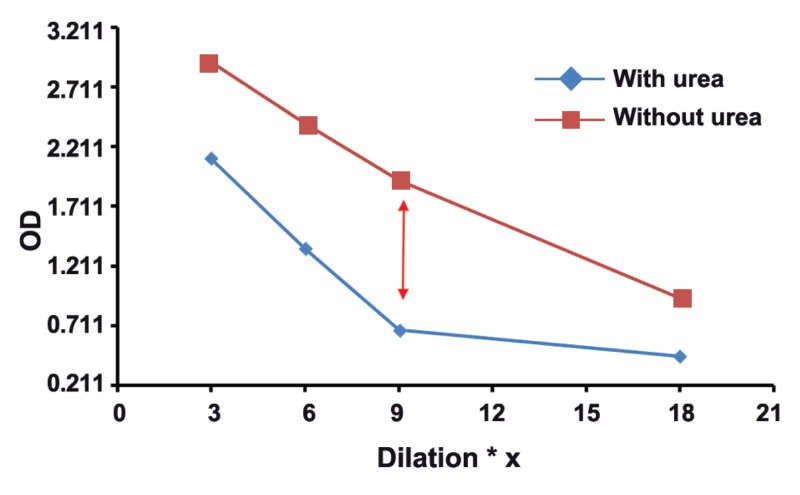
Borderline avidityToxo IgG300-399 Iu/mL.

**Fig 8:**
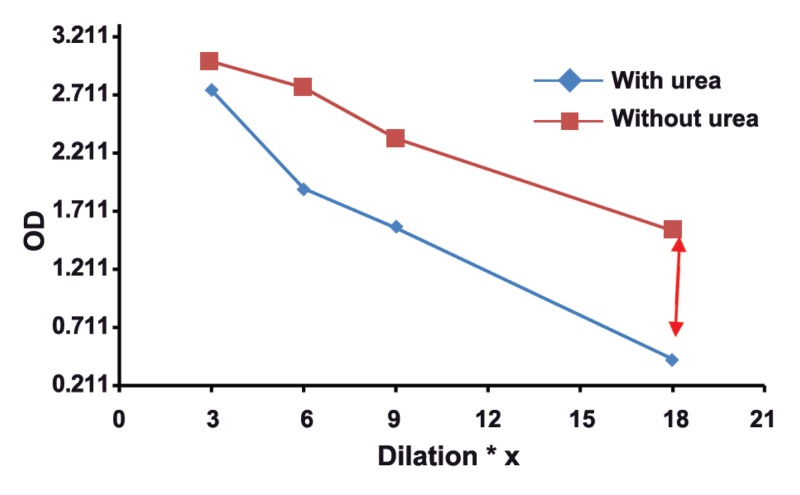
Borderline avidityToxo IgG>400 Iu/mL.

## Discussion

Avidity test for toxoplasmosis was described by Hedman in 1989. Varios companies then produced commercial kits in order to routinize the test. Almost in all kits, the avidity test is used on a single serum sample, and if a good method is not be selected, false toxoplasmosis results followed by low avidity value may occure during the first months of pregnancy (13). Serological diagnosis of toxo IgG and IgM from a single sample can not clearly identify acute infection from chronic one. The resaon is that IgM antibodies tend to remain for years after a primary infection (which emerge early infection), even with high titers (14). Toxo IgM may be stable from six months to 12 years (15-17), so applying an avidity test is the best way to differentiate acute infection from a chronic one. If the characteristics of avidity test reduce, a challenge in diagnosis and treatment of toxoplasmosis may occur. Terefore, by improing avidity method at different concentrations of 100 to 600 iu/ml, we can elevate its sensitivity and specifity.

The exact time of occurance of Toxoplasmic infection is a frequent and difficult challeng in laboratories handling sample from pregnant women, particularly the treatment must start as early as possible to protect the fetus (7, 18). In this study, we investigated the changes for improving the methods of avidity testing to obtain an appropriate serum dilution and to get actual results far from the false negative.

As it was described in method, serum of patients were prepared in different dilutions, and were tested by various commercial kits . The study showed that a proper index of avidity occurred at a certain point of dilution, at which it would have a high specificity (Figs 1-Fig 8). Some of theses commercial kits have been predicted two limiting dilutions for avidity index (13).

These kits are prior to those in which only a single sample is used, but the present study showed that two dilutions of serum samples are not sufficient. The ranges of low avidity and borderline are important, so different studies showed the following wide ranges for them: platelia kit <40% (19) , DIESSE and TESTLINE kit <30% (13),Vidas kit <20 % (13, 15), and liesenfeld study <50% (20). It is because of performance of avidity test on a single sample.

The mesurement of IgG avidity has shown its power in various clinical settings, especialy in situations where timing and differentiation of primary and secoundary infections are crucial (19). However, by improving the method of measuring IgG avidity in mean and high concentrations, the power of differentiation of this test may be increased.

Nowadays, several commercial kits are available for measuring of toxo IgG avidity. There are not many available published cross-evaluations of their diagnostic performances. A high assay modified in-house for avidity determination was observed with immunocompromised patients and healthy adults (21). In another study, a poor correlation has been shown among three commercial IgG-avidity assays (22). The following reports, however, have criticized important points (21). 

## Conclusion

High concenteration of Toxo IgG affects the results of avidity. In our study, the best point of dilution for a good avidity, in both high and borderline avidities, is marked with arrows in figures 1-8. This study revealed that improved methods of measuring Toxo avidity IgG has got a great importance.
